# Reduced regional flow in the left ventricle after anterior acute myocardial infarction: a case control study using 4D flow MRI

**DOI:** 10.1186/s12880-019-0404-7

**Published:** 2019-12-30

**Authors:** Philip A. Corrado, Jacob A. Macdonald, Christopher J. François, Niti R. Aggarwal, Jonathan W. Weinsaft, Oliver Wieben

**Affiliations:** 1 Department of Medical Physics, University of Wisconsin-Madison, 1111 Highland Ave, Madison, WI 53705 USA; 2 Department of Radiology, University of Wisconsin-Madison, 600 Highland Ave, Madison, WI 53792 USA; 3 Department of Medicine, University of Wisconsin-Madison, 600 Highland Ave, Madison, WI 53792 USA; 4 Departments of Medicine and Radiology, Weill Cornell Medical College, 520 East 70th Street, Starr Pavilion, 4th Floor, New York, NY 10021 USA; 5 Departments of Medical Physics and Radiology, University of Wisconsin-Madison, 1111 Highland Ave, Madison, WI 53705 USA

**Keywords:** Phase contrast, 4D flow, Myocardial infarction, Left ventricle

## Abstract

**Background:**

Acute myocardial infarction (AMI) alters left ventricular (LV) hemodynamics, resulting in decreased global LV ejection fraction and global LV kinetic energy. We hypothesize that anterior AMI effects localized alterations in LV flow and developed a regional approach to analyze these local changes with 4D flow MRI.

**Methods:**

4D flow cardiac magnetic resonance (CMR) data was compared between 12 anterior AMI patients (11 males; 66 ± 12yo; prospectively acquired in 2016–2017) and 19 healthy volunteers (10 males; 40 ± 16yo; retrospective from 2010 to 2011 study). The LV cavity was contoured on short axis cine steady-state free procession CMR and partitioned into three regions: base, mid-ventricle, and apex. 4D flow data was registered to the short axis segmentation. Peak systolic and diastolic through-plane flows were compared region-by-region between groups using linear models of flow with age, sex, and heart rate as covariates.

**Results:**

Peak systolic flow was reduced in anterior AMI subjects compared to controls in the LV mid-ventricle (fitted reduction = 3.9 L/min; *P* = 0.01) and apex (fitted reduction = 1.4 L/min; *P* = 0.02). Peak diastolic flow was also lower in anterior AMI subjects compared to controls in the apex (fitted reduction = 2.4 L/min; P = 0.01).

**Conclusions:**

A regional method to analyze 4D LV flow data was applied in anterior AMI patients and controls. Anterior AMI patients had reduced regional flow relative to controls.

## Background

Acute myocardial infarction (AMI) is widespread [1] and has high mortality and morbidity [2]. AMI alters left ventricular (LV) hemodynamics, resulting in increased left ventricular volumes and decreased left ventricular ejection fraction (LVEF) – both powerful prognostic indicators post-AMI [3]. A common complication of AMI is left ventricular thrombus (LVT) - a causal substrate for stroke [4]. In a prospective study of 201 AMI patients, LVT were identified in 8% of all subjects and in 15% of those with anterior infarctions using cardiac magnetic resonance (CMR) within 30 days of infarction, with all thrombi located in the LV apex [5]. The pathogenesis of LVT is caused by a combination of blood stasis, endothelial injury and hypercoagulability, often referred to as Virchow’s triad [6].

Velocity-sensitive imaging offers the opportunity to investigate blood stasis in the post-AMI left ventricle, shedding light on the mechanisms behind high rates of LVT. Several studies to date have investigated LV hemodynamics after myocardial infarction (MI). In a prospective study using two-dimensional (2D) Doppler echocardiography in 104 AMI patients, Dantzig et al. found that abnormal flow (defined as the presence of rotating flow in the apex and/or vortex ring formation) was independently predictive of LVT [7]. However, Doppler echocardiography is limited in spatial coverage (imaging planes are restricted by patient anatomy) and non-specific velocity direction encoding. Another study, using time-resolved, three-dimensional (3D) phase contrast MRI with 3-directional velocity encoding (“4D flow MRI” [8, 9]) in 48 patients with acute or chronic myocardial infarction (MI), found reduced LV kinetic energy in MI patients compared to age/sex-matched controls [10].

While these studies demonstrate that MI alters LV hemodynamics, 4D flow MRI has not yet been applied to look specifically at regional flow differences in anterior AMI patients (the population most relevant to apical LVT risk). Since post-MI LVT typically localizes in the LV apex, we hypothesize that the apex will be most acutely affected by post-anterior-AMI flow reductions. The purpose of this study was to investigate differences in regional intraventricular flow in the LV base, mid-ventricle, and apex between anterior AMI patients and healthy controls using 4D flow MRI.

## Methods

### Study population

Anterior AMI patients were scanned prospectively with 4D flow MRI as an adjunct to their clinical CMR exams performed in 2016–2017. These patients were compared against historical controls from a previously reported, 2010–2011 study [11]. This study was approved by the Institutional Review Board and was compliant with the Health Insurance Portability and Accountability Act. Written informed consent was obtained from all subjects. Inclusion criteria for anterior AMI patients were hospitalization and revascularization for AMI with the left anterior descending artery or left main coronary artery identified as the culprit vessel by coronary angiography, and a clinically ordered CMR exam. Exclusion criteria were contraindications to MRI or gadolinium-based contrast agents. Twelve patients with anterior AMI were recruited. Exclusion criteria for control subjects were standard contraindications to MRI and to gadolinium-based contrast agents, high cardiovascular risk factors (body mass index > 30, history of smoking, diabetes, or hypertension), and drugs affecting cardiovascular function. Data from 19 control subjects were included in the analysis, for a total of 31 scans analyzed.

### MRI acquisition

CMR examinations in controls were performed in a 3.0 T scanner (MR750, GE Healthcare, Waukesha, WI). CMR examinations in AMI subjects were acquired on 1.5 T (MR450w or HDxt, GE Healthcare, Waukesha, WI; *N* = 9) and 3.0 T (MR750 or MR750w, GE Healthcare, Waukesha, WI; *N* = 3) scanners. The choice of field strength was based on the clinical availability of the scanners. The CMR protocol included a short-axis bSSFP cine acquisition to segment the LV cavity & compute LV strain, 4D flow imaging for velocity mapping, and short-axis late gadolinium enhancement (LGE; only in AMI patents) imaging to measure infarction size. 4D flow data was acquired with PC VIPR, a three-dimensional radially-undersampled, three-directionally velocity encoded technique [12, 13], with a scan duration of 9–14 min including respiratory gating efficiency. Each set of 4D flow data was reconstructed in two ways: a high-resolution image set was reconstructed using a gridding technique and used to measure through-plane flow and intraventricular KE, and a low-resolution, low-noise image set was reconstructed using compressed sensing with a spatial-wavelet-transform L1-norm penalty (λ = 0.01) in order to separate LV flow into different compartments by tracking flow pathlines. This separate reconstruction was used for pathline tracking because this method is sensitive to noise due to compounding errors in pathline integration. Intravenous contrast was administered to all subjects prior to 4D flow imaging. AMI patients received 0.15 mmol/kg of gadobenate dimeglumine (Multihance; Bracco, Milan, IT) and controls received 0.03 mmol/kg of gadofosveset trisodium (Ablavar; Lantheus, Billerica, MA, USA). Table 1 shows the MRI acquisition parameters.

**Table 1:** MRI acquisition parameters

Parameter	2D Short-Axis bSSFP	4D flow (PC VIPR)	2D LGE Scar Imaging
ECG type	prospectively gated	retrospectively gated	triggered (diastolic phase)
Cine Frames	20	20	–
Respiratory Motion Strategy	breath holding	free-breathing	breath holding
Repetition Time (ms)	3.1–4.1	5.8–8.4	4.0–4.8
Echo Time (ms)	1.1–1.2	2.0–2.5	1.4–2.2
Flip Angle (degrees)	45	8–12	20–45
Inversion Time (μs)	–	–	183–400
Field of View (cm^2^)	39 × 39	–	35 × 35
Slice Thickness (mm)	8	–	8
Imaging Volume (cm^3^)	–	32 × 32 × 20	–
Acquired Spatial Resolution (mm)	1.74 × 1.74	1.25 × 1.25 × 1.25	2.03 × 2.03
Reconstructed Spatial Resolution (mm)	1.25 × 1.25	High resolution reconstruction:1.25 × 1.25 × 1.25 Low resolution reconstruction: 2.5 × 2.5 × 2.5	1.37 × 1.37
Velocity Encoding (cm/s)	–	100–150	–

Note—Reported values are the ranges (minima and maxima) for each parameter. Parameters were tailored to each individual’s anatomy and the field strength used. Two sets of images were reconstructed for each 4D flow acquisition: a high-resolution image set used to compute flow and ventricular KE, and a low-resolution image set used to separate LV flow components by tracking pathlines

### Image analysis

The LV cavity was segmented at each time frame on short-axis bSSFP images (including outflow tract, excluding papillary muscles) using the software Segment (Medviso, http://segment.heiberg.se; v2.0 R5399) [14]. LV end diastolic volume (EDV), end systolic volume (ESV), stroke volume (SV), and ejection fraction, were calculated from the endocardial borders. Global longitudinal strain (GLS) was computed from long-axis bSSFP images, and regional radial and circumferential strain were computed from short-axis bSSFP images using one slice each in the base, mid-ventricle, and apex in Segment using feature tracking [15].

**Fig. 1:**
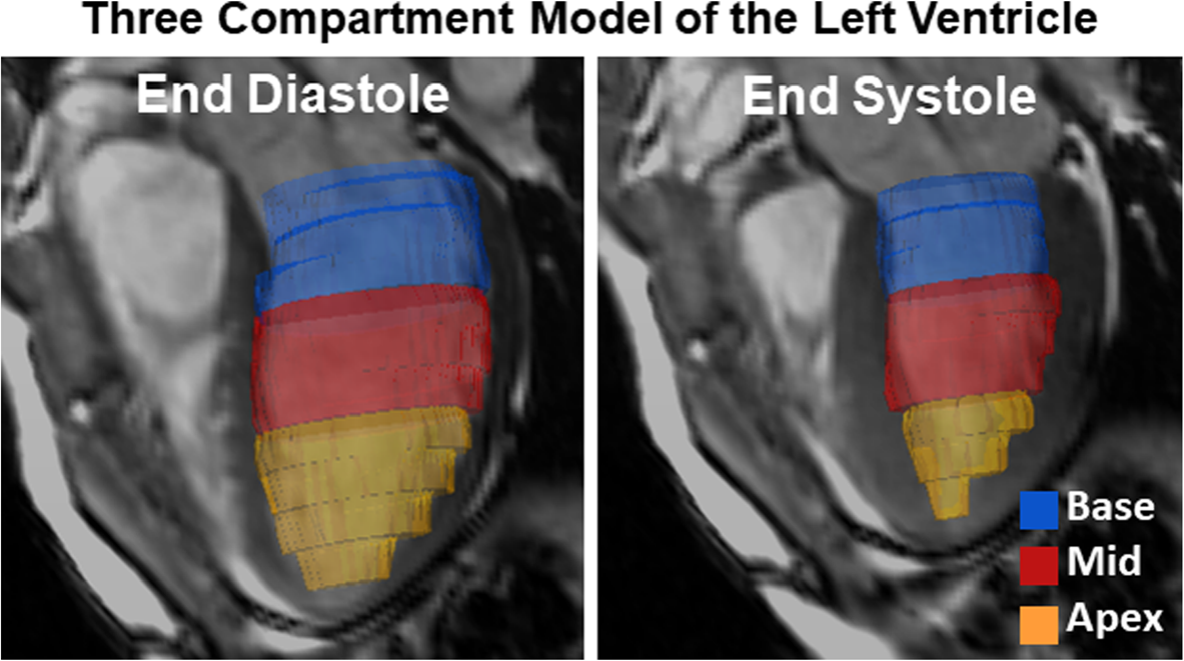
3D visualization of time-resolved LV segmentation produced from cine short-axis bSSFP images, displayed at end diastole (left) and end systole (right). The LV cavity was subdivided into three regions along the LV long axis for regional flow analysis: base (blue), mid-ventricle (red), and apex (orange). A co-registered long-axis bSSFP image slice is overlaid for viewing convenience. These visualizations were generated using Ensight 10.0 (Ansys, Canonsburg, PA)

4D flow data background phase errors were corrected by fitting a 3rd order polynomial to static tissue phase. Subsequently, 4D flow data was registered to the short-axis bSSFP dataset using the Advanced Normalization Toolkit [16, 17]. Proper registration was confirmed by overlaying the images in ITK-SNAP [18]. Each slice in the LV segmentation was assigned to one of three equal-length segments divided along the LV long axis: base, mid-ventricle, and apex (Fig. 1). Through-plane flow was computed for each slice and time point by multiplying the average through-plane velocity component in the LV with the cross-sectional area of the LV. Through-plane flow in each LV region was computed as the average through-plane flow for each slice in that region. Peak systolic and diastolic flows were defined for each region as maximum positive and negative flows, with the positive through-plane direction running from the apex to the base.

Average KE was computed by summing the KE contributions for all voxels in the LV and averaging over time:

$$ {KE}_{avg}=\frac{1}{N_t}\sum \limits_{time}\sum \limits_{vox els}{KE}_{vox}=\frac{1}{N_t}\sum \limits_{time}\sum \limits_{vox els}\frac{1}{2}{\rho}_{blood}{V}_{vox}{v_{vox}}^2 $$

Where N_t_ is the number of cardiac time frames, ρ_blood_ is the density of blood (1.06 g/cm^3^), V_vox_ is the voxel volume, and v_vox_ is the velocity magnitude. KE_avg_ was then indexed to EDV (KEi_EDV_) as in Garg et al. [10]. KE was not computed on a regional scale because the squared velocity term results in a measurement dominated by high-velocity voxels, which is therefore highly sensitive to noise in slow-flow regions (such as the LV apex) with a full-ventricle high velocity encoding imaging approach.

The distribution of different LV flow components was determined in all subjects using the method of Eriksson et al. [19]. Blood pathlines were emitted from the LV blood volume and traced forwards and backwards in time from end diastole until end systole, thus including the entire cardiac cycle. Pathlines were computed by integrating the velocity field using a 4th order Range-Kutta numerical integration through time. Pathline location was used to separate the pathlines into four different components of flow: Direct Flow (blood that enters the LV during diastole and leaves the LV during systole in the analyzed heartbeat), Retained Inflow (blood that enters the LV during diastole but does not leave during systole in the analyzed heartbeat), Delayed Ejection Flow (blood that starts and resides inside the LV during diastole and leaves during systole), and Residual Volume (blood that resides within the LV for at least two cardiac cycles). Pathlines passing through the ventricle wall (either entering or leaving the LV through the mid-ventricle or apical regions) were excluded from analysis. The fraction of EDV containing pathlines from each compartment was computed for all subjects.

All flow computations were performed in Matlab (R2018a, The Mathworks Inc., Natick, Massachusetts, USA). All bSSFP and 4D flow images were analyzed by PAC (3 years of CMR analysis experience). Kim’s method [20] was used on LGE images to compute infarct size as follows: each segment in the 17-segment AHA myocardial model was scored for infarction transmurality using a 5-point scale (0 = no infarction, 1 = 0–25%, 2 = 25–50%, 3 = 50–75%, 4= > 75% transmurality), the scores were averaged, and the result was divided by four. The images were scored by consensus of two radiologists with expertise in cardiothoracic imaging (reader 1, 17 years of experience; reader 2, 5 years of experience). Disagreements were handled by consulting a third radiologist. The LGE readers were blinded to the results of the flow analysis and vice-versa.

### Statistical analysis

Demographic and traditional CMR measures (LV function and strain) are presented as mean ± standard deviation and were compared between AMI patients and controls using independent sample t-tests. The proportion of male subjects was compared between groups using a chi-squared test. The 11 LV flow parameters (peak systolic and diastolic flow in each region, global KEi_EDV_, and the percentage of LV flow in each of the 4 compartments) are not assumed to be normally distributed and are presented as median ± inter-quartile range (IQR). Multivariable linear regression was used to test if there were differences in the 11 LV flow parameters between anterior AMI patients and controls, while adjusting for age, sex and heart rate differences between the groups as covariates. For each LV flow parameter, a multivariable linear regression model was fitted with LV flow parameter as outcome, group of subjects (AMI patient or control) as predictor variable, and age, sex, and heart rate as covariates in the model. Model diagnostics were performed and no serious violations of the assumptions of the linear models were found. Regional LV through-plane flow parameters were correlated with heart rate and traditional CMR measures (infarct size, SV, CO, EF, EDV, GLS, and regional radial and circumferential strain) using Spearman’s rank correlation test. Regional through-plane flow and regional strain were compared on a region-by-region basis (i.e. flow in the LV base was compared with strain in the LV base). Multivariable regression modeling was performed in R version 3.5.2. All other analysis was performed in MATLAB. A significance level of 0.05 was used for all tests.

## Results

### Study population

Ten of the 12 anterior AMI subjects (83%) had ST-segment elevation. The mean interval between AMI and CMR was 3.7 days with a range of 1–13 days. Peak measured troponin-I levels in AMI subjects had a mean of 119.1 ng/mL and a range of 0.08–309.9 ng/mL. The mean percentage of myocardium infarcted was 26% with a range of 4–53%. Three AMI subjects (25%) had a left ventricular thrombus present at the time of imaging. Table 2 shows demographics and traditional CMR measures for healthy controls and AMI subjects.

**Table 2:** Demographics, LV function, and LV strain measures in all subjects

	Controls (*n* = 19)	Anterior AMI Patients (*n* = 12)	*P*-value
Sex (male, n)	10	11	**0.02**
Age (years)	40 ± 16	66 ± 12	**2E-05**
Weight (kg)	76 ± 11	86 ± 25	0.23
Heart rate (bpm)	60 ± 10	79 ± 15	**0.002**
Stroke volume (mL)	95 ± 16	65 ± 21	**5E-04**
Cardiac output (L/min)	5.6 ± 0.9	5 ± 1.1	0.10
End diastolic volume (mL)	141 ± 24	149 ± 32	0.46
Ejection fraction (%)	68% ± 6%	44% ± 10%	**2E-06**
Radial strain (%)			
Base	31% ± 9%	33% ± 14%	0.73
Mid-ventricle	42% ± 9%	28% ± 18%	**0.02**
Apex	36% ± 18%	14% ± 20%	**0.01**
Circumferential strain (%)			
Base	−18% ± 2%	−12% ± 4%	**1E-04**
Mid-ventricle	−19% ± 3%	−10% ± 5%	**2E-05**
Apex	− 21% ± 4%	− 6% ± 5%	**5E-08**
Longitudinal strain (%)	−16% ± 2%	− 7% ± 3%	**1E-07**

Note—Reported values are mean ± 1 standard deviation. *P*-values were computed using two-sample t-tests. Boldface signifies *P*<0.05

### LV flow measurements

**Fig. 2:**
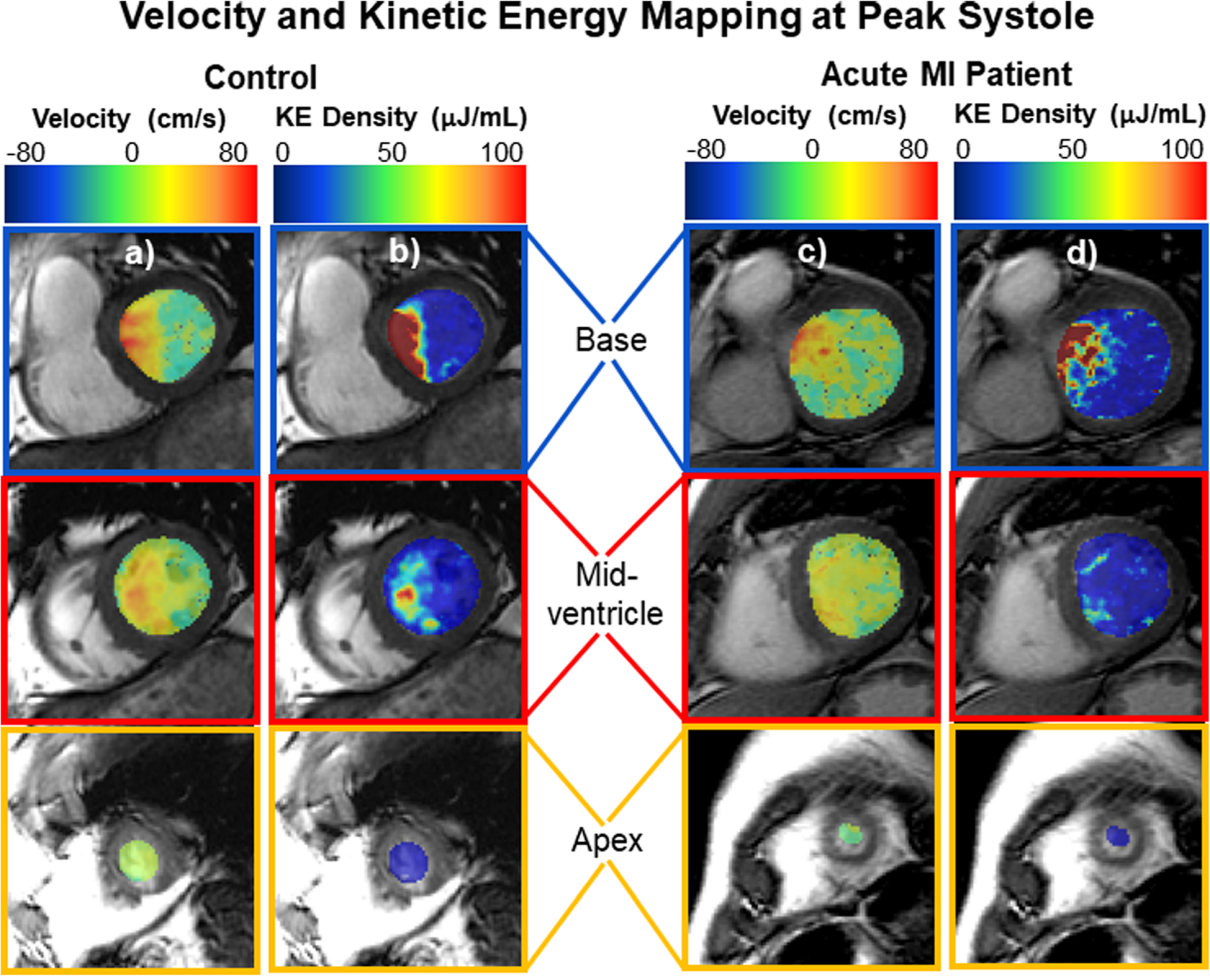
Maps of through-plane velocity (**a** & **c**) and kinetic energy density (**b** & **d**) in selected left ventricular slices at peak systole in a control subject (left, **a** & **b**, 73-year-old male) and in an acute myocardial infarction patient (right, **c** & **d**, 73-year-old male). Positive though-plane velocities represent the apex-to-base direction. Velocities are lower in the mid-ventricle and apex of the acute MI subject compared to the control. The maps were created by registering 4D flow data to short-axis cine bSSFP images. The overlays were generated with ITK Snap

**Fig. 3:**
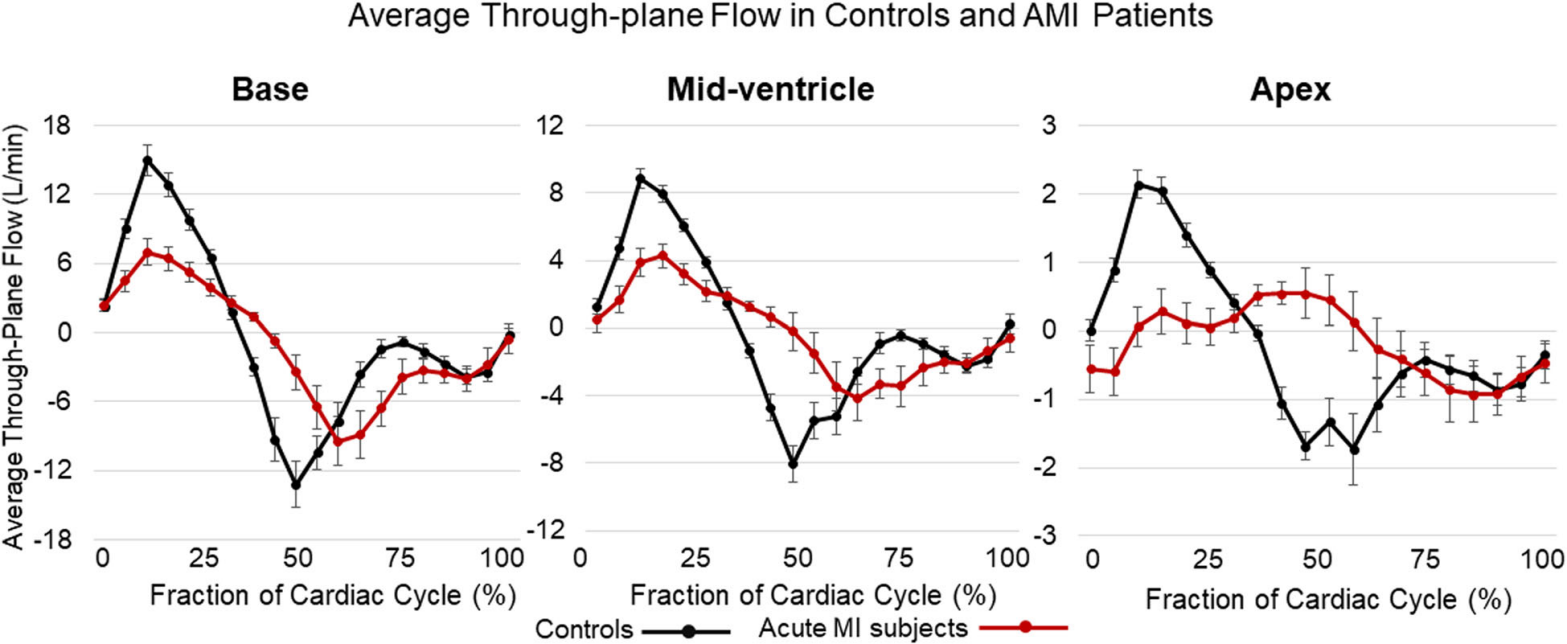
Group-averaged through-plane flow curves in the left ventricular base (left), mid-ventricle (center), and apex (left) for short-axis planes. The positive direction runs from the apex to the base. Error bars represent the standard error for each group

**Fig. 4:**
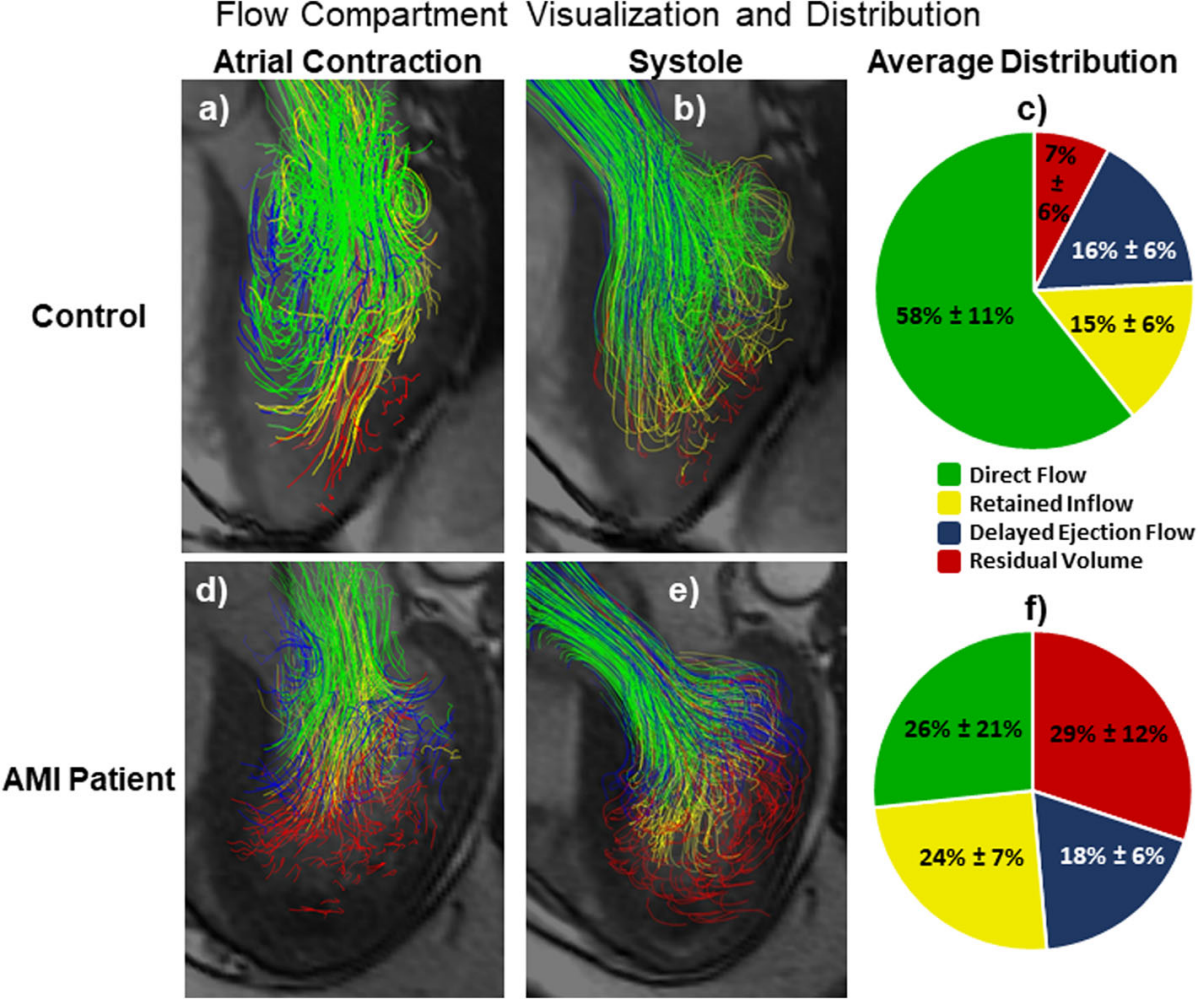
Flow pathline visualization showing different flow compartments in a control (**a**&**b**, 48yo M) and an anterior AMI patient (**d**&**e**, 66yo M). The right column shows group-averaged flow component distributions in **c**) controls and **f**) anterior AMI patients. Reported values are median ± interquartile range. Green = Direct Flow: Blood that enters the LV during diastole and leaves during systole of the same heartbeat. Yellow = Retained Inflow: Blood that enters the LV during diastole but does not leave during systole. Blue = Delayed Ejection Flow: Blood that starts inside the LV during diastole and leaves during systole. Red = Residual Volume: Blood that resides within the LV for at least two cardiac cycles

For all subjects analyzed, 4D flow data was successfully registered to short-axis cine images for through-plane velocity mapping. Figure 2 shows examples of through-plane velocity and KE mapping at peak systole in a representative control subject and in a representative subject with an anterior AMI. Compared to the control subject, the anterior AMI subject has lower through-plane velocities in all LV regions. In both subjects, the KE map is dominated by regions near the left ventricular outflow tract. Figure 3 shows average through-plane flow-time curves in each region for anterior AMI patients and controls. Figure 4 shows representative visualizations of flow compartment analysis in a control (a, b) and AMI subject (d, e). The AMI subject had a lower fraction of pathlines in the direct flow compartment and a higher fraction of pathlines in the residual volume compartment. Subpanels c) and f) show group-average pie charts of the distribution of flow among compartments. Among all subjects, the median number of pathlines passing through the ventricular wall (and therefore discarded) was 24%. Table 3 shows the average 12 LV flow parameters in controls and anterior AMI patients, along with *P*-values for the comparison. Compared to controls, anterior AMI subjects had significantly lower through-plane flow in the mid-ventricle at peak systole and in the apex at peak systole and diastole. There were no significant differences in KEi_EDV_ measures between groups. Compared to controls, anterior AMI subjects had significantly less direct flow and significantly more retained inflow, delayed ejection flow, and residual volume.

**Table 3:** LV flow parameters in controls and anterior AMI patients

Flow Parameter	Region	Controls (*n* = 19)	Anterior AMI (*n* = 12)	*P*-value	Δ_MI_	95% CI
Peak Systolic Flow (L/min)	Base	16.2 ± 5.4	8.4 ± 6.8	0.28	−2.1	[−5.8, 1.6]
	Mid-ventricle	8.5 ± 3.6	5.5 ± 3	**0.01**	−3.9	[− 6.4, − 1.4]
	Apex	2.2 ± 1	1.4 ± 1.4	**0.02**	−1.4	[−2.6, − 0.3]
Peak Diastolic Flow (L/min)	Base	18.6 ± 6.9	13.3 ± 4.7	0.71	1.1	[−4.6, 6.8]
	Mid-ventricle	12 ± 4.3	9.1 ± 4.3	0.35	−1.6	[−4.9, 1.7]
	Apex	3.1 ± 1.5	2 ± 1.1	**0.004**	−2.4	[−4.0, − 0.9]
Average KEi_EDV_ (μJ/mL)	Whole LV	11.3 ± 2.7	16.6 ± 11.9	0.79	− 0.9	[−8.0, 6.1]
Direct Flow %	Whole LV	58 ± 11	26 ± 21	**4E-08**	−41	[−52, −31]
Retained Inflow %	Whole LV	15 ± 6	24 ± 7	**.0003**	12	[7, 18]
Delayed Ejection Flow %	Whole LV	16 ± 6	18 ± 6	**0.005**	8	[3, 13]
Residual Volume %	Whole LV	7 ± 6	29 ± 12	**6E-06**	21	[14, 28]

Note—Reported values are median ± interquartile range (IQR). *P*-values were computed using multivariable linear regression models with age, sex, and heart rate as covariates. Bold text represents a significant *P*-value. Δ_MI_ is the expected difference in the LV flow parameter due to the anterior AMI. 95% CI is the 95% confidence interval for Δ_MI_

KEi_EDV_: Kinetic energy indexed to end diastolic volume

### Correlation of LV flow parameters with infarct size and traditional CMR measures

Spearman’s rank correlation coefficient was used to determine the correlation of heart rate (HR) and traditional CMR metrics (infarct size, LV SV, LV CO, LV EF, LV EDV, GLS, and regional radial and circumferential strain) with through-plane flows in each region at peak systole and diastole. Table 4 shows the *P*-values for the correlation coefficient, with the coefficient in parentheses for significant correlations. Peak basal systolic through-plane flow was negatively correlated with HR and peak basal circumferential strain and positively correlated with SV and EF. Peak mid-ventricular through-plane flow was also positively correlated with EF. There were no significant correlations with through-plane flow in the apex or with any diastolic flow measures.

**Table 4:** P-values for correlation of regional LV flow parameters with traditional CMR parameters in anterior AMI patients

	Peak Systolic Flow (L/min)			Peak Diastolic Flow (L/min)		
	Base	Mid-ventricle	Apex	Base	Mid-ventricle	Apex
Infarct size	0.81	0.19	0.66	0.36	0.14	0.3
Heart rate	**0.02 (−0.64)**	0.14	0.28	0.85	0.13	0.17
Stroke volume	**0.03 (0.63)**	0.11	0.28	0.72	0.57	0.35
Cardiac output	0.46	0.99	0.22	0.59	0.77	0.83
Ejection fraction	**0.001 (0.84)**	**0.03 (0.62)**	0.19	0.38	0.79	0.24
End diastolic volume	0.97	0.84	0.23	0.9	0.56	0.66
Global peak longitudinal strain	0.13	0.35	0.67	0.8	0.57	0.33
Regional peak radial strain	0.08	0.2	0.6	0.67	0.96	0.42
Regional peak circumferential strain	**0.02 (−0.69)**	0.15	0.67	0.65	0.92	0.43

Note—Shown are P-values for spearman’s correlation coefficient. For P-values <0.05, the correlation coefficient is included in parentheses, and the box is in boldface text. Through-plane flow and strain were compared on a region-by-region basis (i.e. flow in the LV base was compared with strain in the LV base)

## Discussion

The focus of this study was to apply 4D flow MRI to compare regional and global LV flow between anterior AMI patients and controls. To do so, through-plane flows in the LV base, mid-ventricle, and apex, as well as global KEi_EDV_ and the fraction of LV flow in each of the 4 flow compartments, were measured in 12 patients and 19 controls. AMI patients had reduced through-plane flow in the apex at peak systole and diastole, and in the mid-ventricle at peak systole compared to controls, even after correcting for age, sex, and heart rate differences between the groups. While the other flow measurements were also lower in patients than in controls, the differences were not significant after correcting for age, sex, and heart rate differences. This finding supports the notion of stasis in the LV apex of anterior AMI subjects contributing to the elevated rates of LVT experienced by this group. The implication of this finding is that 4D flow MRI of the LV apex may be a valuable tool in larger studies on post-AMI LVT risk assessment. While the more traditional hemodynamic metric, LVEF, was also lower in patients than in controls, this measure does not offer insights into the location of flow impairment. This study did not find the reduction in KEi_EDV_ that Garg et al. found in a cohort of 48 MI subjects [10]. This is likely caused by the smaller sample size of this study and the use of different scanners for different subjects, which we elaborate on in the limitations paragraph below. Flow compartment analysis revealed a marked shift away from blood entering and leaving the LV in one heartbeat (direct flow) towards blood starting and residing in the LV for > = 2 heartbeats (residual volume) in anterior AMI patients. This increase in residual volume is consistent with the decreased through-plane flow in the apex (since residual flow pathlines are typically located in or near the apex) and with the notion of increased stasis post MI. Our control data have similar flow rates to those reported in 1995 [21] and similar KE values to those reported in 2015 [22] and 2016 [23] in healthy controls. Our control data however displays a greater fraction of flow in the direct flow compartment and less in the residual volume compartment than Eriksson et al. found in 2010 [19] and 2013 [24]. We attribute this to differences in acquisition such as k-space trajectory (radial vs. cartesian), spatial resolution (2.5 × 2.5 × 2.5mm^3^ vs. 3x3x3mm^3^), number of cardiac frames (20 vs. 40), and scan duration (9–14 min. vs. 16–57 min.), although it is unclear how this difference led to the observed differences in flow compartment distribution. Despite the fact that our baseline LV flow compartment distribution in controls differs from that in the literature, our finding of flow compartment shifts in AMI patients relative to controls is valid since all of the 4D flow data presented herein was acquired with the same sequence.

Peak systolic flow in the LV base was negatively correlated with heart rate and circumferential strain and positively correlated with stroke volume and ejection fraction. The negative correlation of peak flow with heart rate may result from compensatory heart rate increases in order to maintain cardiac output in the infarcted individuals with reduced intraventricular flow. The negative correlation with circumferential strain means that individuals with the largest magnitude of strain had the highest flow in the LV base (because circumferential strain is negative). Ventricles that deformed more where able to push more blood through at peak systole. This correlation may not have been significant in other LV regions due to sample size limitations. The positive correlations of peak systolic intraventricular flows with SV and EF are expected since SV is the total flow over one cardiac cycle at the aortic valve, and EF is directly related to SV. The lack of significant correlation between the LV flow metrics and infarct size could be attributed to the small sample size of the MI group.

### Limiations

One limitation of this pilot study is the relatively low number of subjects in each cohort. Additionally, 4D flow data in patients was collected as an adjunct to clinical CMR exams. Accordingly, multiple scanners (1.5 T and 3 T) were used depending on clinical availability. As a result, imaging parameters and image quality slightly varied between patients, and quality might have been compromised in comparison to controls (all imaged at 3 T). The use of 1.5 T scanners for some AMI patients limited sensitivity but did not create a systematic bias in AMI patient flow versus controls, as data from Lotz et al. show that the use of 1.5 T versus 3 T scanners reduces flow measurement precision but not accuracy [25]. Likewise, the difference in contrast agent used between the two groups may have created different blood T1 constants and therefore velocity-to-noise ratios in the two groups but would not create a bias between the through-plane flow measurements in each group. The use of different scanners and contrast agents may have affected our KE measurements, however, as erroneously measured high-velocity voxels can dominate the KE sum since it is derived from velocity squared. Moreover, the velocity encoding parameter selection at 100–150 cm/s may have been higher than optimal for detecting differences in low velocities (stasis), such as those present in the apex. However, a lower velocity encoding parameter would have resulted in aliased and incorrect flow measurements in the LV base and mid-ventricle. Further studies investigating and validating the optimal 4D flow acquisition settings in the context of AMI are warranted. While the relatively low sample size, increased noise in patient data, and perhaps suboptimal VENC setting may have reduced the power of this study, this study did shed light on which regions of the intra-LV flow field are most affected after anterior AMI. Another limitation of this study is the comparison of the prospectively acquired AMI cohort with the control cohort from a previous study. As such, there were differences between the AMI and control groups such as age, sex and heart rate. While our model corrected for differences in age, sex, and heart rate between the groups, we acknowledge that a prospectively matched study would provide stronger evidence of post-AMI flow reductions.

## Conclusions

This study provided a methodology for the regional analysis of 4D LV flow data and applied that methodology in anterior AMI patients and healthy controls. AMI subjects demonstrated reduced through-plane flow in the LV, even after correcting for age, sex, and heart rate differences. Further investigation is necessary to determine whether regional LV flow has predictive value in the post-AMI population.

## Data Availability

The datasets analyzed during the current study are available from the corresponding author on reasonable request.

## Abbreviations

2D: two-dimensional
3D: three-dimensional
4D flow: Four-dimensional flow
AMI: Acute myocardial infarction
bSSFP: balanced steady-state free precession
CMR: Cardiovascular magnetic resonance
ECG: Electrocardiogram
FA: Flip angle
FOV: Field of view
LGE: Late gadolinium enhancement
LV: Left ventricle
LVEF: Left ventricular ejection fraction
LVT: Left ventricular thrombus
MI: Myocardial infarction
SNR: Signal-to-noise Ratio
TE: Echo time
TR: Repetition time
VNR: Velocity-to-noise Ratio
